# Keys to successful implementation of routine symptom monitoring in head and neck oncology with “Healthcare Monitor” and patients' perspectives of quality of care

**DOI:** 10.1002/hed.26425

**Published:** 2020-08-18

**Authors:** Emilie A. C. Dronkers, Robert J. Baatenburg de Jong, Egge F. van der Poel, Aniel Sewnaik, Marinella P. J. Offerman

**Affiliations:** ^1^ Department of Otorhinolaryngology and Head and Neck Oncology Erasmus University Medical Center Cancer Institute Rotterdam The Netherlands

**Keywords:** head and neck cancer, patient‐reported outcome measures, quality assurance, quality of life, value‐based health care

## Abstract

**Background:**

Value‐based health care is increasingly used to facilitate a systematic approach during follow‐up of patients. We developed Healthcare Monitor (HM): a structure of electronic patient‐reported outcome measures (ePROs) for the longitudinal follow‐up of head and neck cancer (HNC) patients. This study shares key lessons from implementation and seeks to provide insight into how patients experience HM.

**Methods:**

We conducted a mixed‐methods study using quantitative data from a nonrandomized retrospective survey of patients who received HM (n = 45) vs standard care (n = 46) and qualitative data from structured interviews (n = 15).

**Results:**

Implementation of HM included significant challenges. Finding common ground among clinicians, administrators, and IT staff was most important. Qualitative findings suggest that patients experienced better doctor‐patient communication and increased efficiency of the consultation using HM. Patients felt better prepared and experienced more focus on critical issues. Quantitative analysis did not show significant differences.

**Conclusions:**

Integration of HM into routine care for HNC patients may have increased patient‐centered care and facilitated screening of symptoms. However, future research is needed to analyze the potential benefits more extensively.

## 1 INTRODUCTION

Value‐based health care is increasingly used to stimulate patient‐centered care and to empower patients during doctor‐patient encounters.^1^, ^2^, ^3^ This concept, which was first described by Michael Porter, claims that improvement in both quality and cost of care can be achieved by understanding and integrating the patient perspective into care.^2^ To help clinicians better understand the patient perspective, use of patient‐reported outcome measurements (PROMs) is recognized as essential.^3^ They are defined as standardized, validated questionnaires completed by patients to measure their perception of their functional well‐being and health status.^4^ Electronic PROMs (ePROs) allow for efficient standardized assessment and improved ease of use in comparison with paper‐based PROMs.^4^, ^5^, ^6^

Understanding the patient's perspective is important during follow‐up of patients with cancer, since doctor‐patient communication can be challenging for both patients and doctors. Patients can have difficulties sharing a complete health status in a short period of time and doctors also need to have good skills to facilitate this process.^7^, ^8^ Physical impairments and psychosocial problems may go undetected and opportunities to intervene can be missed.^9^, ^10^ By using ePROs, patients might actively participate in their own care and clinicians identify critical issues, improving patient management.^4^, ^6^, ^9^, ^11^, ^12^, ^13^, ^14^

ePROs focus on physical problems, psychosocial problems, and/or the impact on global health‐related quality of life (HRQoL).^4^ They provide data detailing the patient's own view on the impact of having cancer and its treatment. Furthermore, ePROs can capture a more holistic view on individual health outcomes. There is evidence from general cancer care that clinical interventions following the routine use of ePROs in clinical practice may improve patients' HRQoL, enhance doctor‐patient communication, and may even lengthen survival, for example, among patients with advanced cancers.^1^, ^12^ ePROs can also play a role in shared decision making.^15^, ^16^ However, monitoring of ePROs alone does not improve patients' outcomes.^8^, ^17^, ^18^ Providing individual feedback to the patient can help to, for example, discuss the need for supportive care.^4^, ^19^, ^20^, ^21^

The ePRO approach is also getting more attention in head and neck cancer (HNC) care.^22^, ^23^ Due to advancements in diagnosis and treatment, the number of HNC survivors have increased.^24^ However, HNC patients often have to deal with treatment‐related side effects that can have an enormous impact on patients' daily life. Some of these side‐effects are immediately noticeable in social settings and can negatively affect HRQoL and increase levels of psychological distress and on the spousal relationship.^25^, ^26^, ^27^ ePROs might support patients in coping with the physical and emotional challenges of HNC by providing themselves and their clinicians better insight into their condition.

In 2013, we developed Healthcare Monitor (HM), an ePRO‐based clinical support system, which uses simple and internationally validated questionnaires regarding HNC to measure physical and psychosocial functioning from day of diagnosis until 5 years after. Since 2015, HM has been structurally embedded in our care for HNC patients.

The overall aim of this study was to provide a first evaluation of HM after implementation. We first review the challenges we experienced during the initial implementation phase (ie, clinical impressions). In addition, we then evaluate patients' experiences with HM in practice and the perceptions of quality of care among patients receiving standard care vs those receiving HM care.

## 2 MATERIALS AND METHODS

### 2.1 HM: description and organizational setting

**FIGURE 1:**
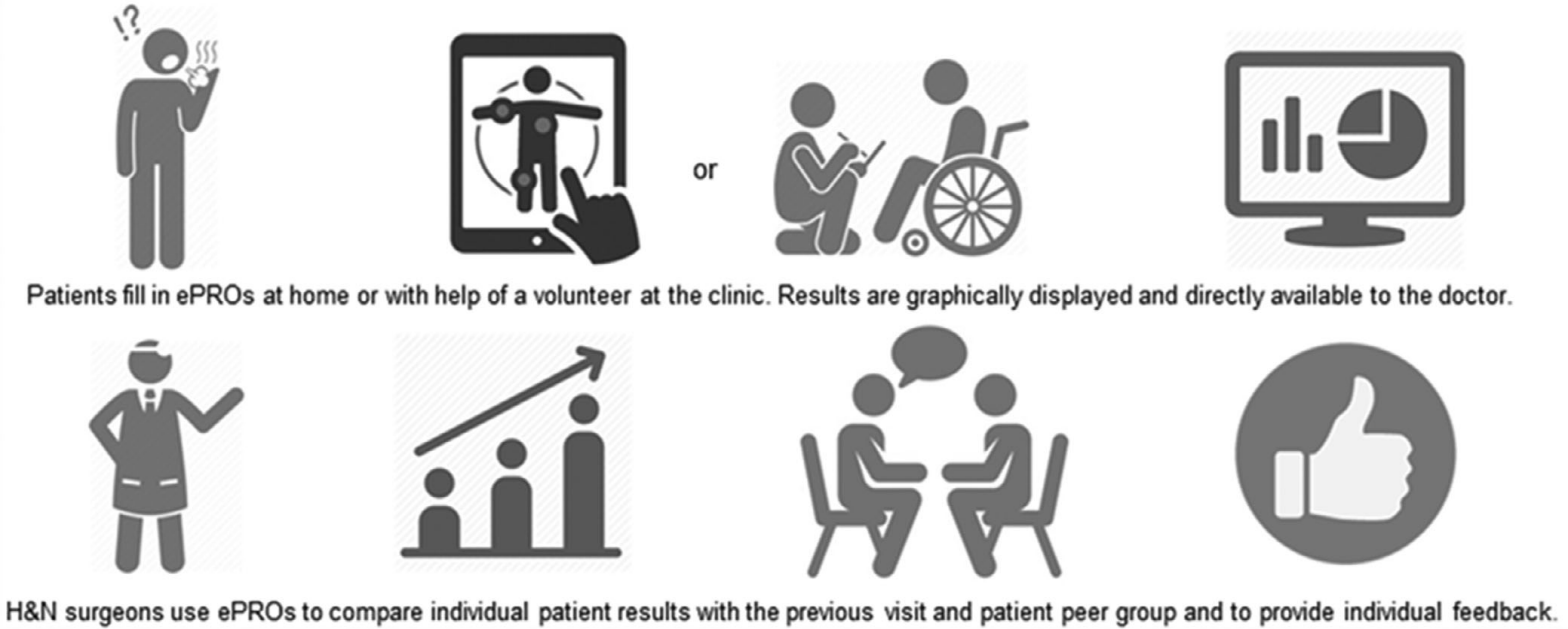
Infographic Healthcare Monitor

The Erasmus Medical Center (Erasmus MC) houses the largest HNC center in the Netherlands with over 600 new patients annually. In 2013, we developed Healthcare Monitor (HM) (see Figure 1). This is an ePRO‐based clinical support system, designed with health care professionals and technology providers for follow‐up and management of HNC patients. Our vision behind the development of HM is threefold:

1. improve overall quality of patientcare,
2. support research in general,
3. improve transparency of health care for the purpose of national registries and audits.

Internationally validated questionnaires (Table 1),^28^, ^29^, ^30^, ^31^, ^32^, ^33^, ^34^ measuring physical problems, psychosocial symptoms, and HRQoL of HNC patients, are routinely collected with HM from the first visit at the day of diagnosis to the final consultation (5 years after end of treatment). A dedicated nurse practitioner counsels patients on the way of working with HM. Patients suffering from disorders affecting cognitive abilities (eg, dementia, Korsakoff syndrome) may be excluded from participation in HM. All patients complete questionnaires before every outpatient clinic visit. They do this either in the comfort of home via Internet or with an iPad at the clinic. In case patients want assistance with filling out the questionnaires, for example, due to illiteracy, trained volunteers are available to help. These volunteers are already part of our specialized outpatient clinic team for HNC patients. They help patients with the logistic procedures during diagnosis.

**TABLE 1:** Generic and specific patient‐reported outcome measurements used in Healthcare Monitor

General questionnaire: items on lifestyle, socioeconomic status, and civil status
EORTC QLQ‐C30: assesses the quality of life of patients with cancer
EORTC H&N35 module: measures the quality of life specifically in patients with head and neck cancer
Hospital Anxiety and Depression Scale (HADS): measures symptoms of anxiety and depression in patients
Eating Assessment Tool (EAT10): assesses swallowing function
Voice Handicap Index (VHI): assesses function of voice and speech
EQ5D‐3L: generic standardized measure of health status

Clinicians have real‐time access to the results that are graphically displayed inside the electronic health record (EHR). Clear graphics help to systematically monitor symptoms and allow clinicians to compare individual patient results during the course of time and with their peer group. Firstly, published norms from questionnaires (Table 1) were used as peer group information.^28^, ^29^, ^30^, ^31^, ^32^ Since 2018, we also evaluate scores from all other HNC patients treated at Erasmus MC as the amount of data is becoming sufficient to make a valid comparison from that time onwards. Scores from HNC patients evaluated at the same point in posttreatment follow‐up care, with the same tumor type and stage are used for this purpose.

The literature shows us that monitoring of symptoms alone is not enough.^35^ Our HM concept therefore includes both monitoring and sharing the results with patients. Clinicians use graphs to provide direct individual feedback to each patient. All patients receive further clinical or diagnostic evaluation or referral to specialty care as needed. HM data and the conversation following the individual feedback can thus further guide health care providers and patients on supportive care needs.

Patients' informed consent to use their data for peer group information and for broader research and benchmark purposes is requested before and after treatment. Since the time between diagnosis and first regular follow‐up visit after treatment can easily reach up to 6 months, we believe it is more conscientious to ask patients again for their consent after treatment.

We started HM with a pilot phase from November 2013 to March 2015 among 260 patients with small (T1‐2) laryngeal carcinoma, treated with (laser) surgery. Patients were included at any point during their diagnostic, treatment or posttreatment trajectory (up to 5 years after diagnosis). Total response was 97% at intake and 90% in follow‐up phase. As a first (nonscientific) evaluation, these patients were asked after their visit at the out‐patient clinic if they thought HM to be an improvement of care and 70% agreed. Physicians also found HM an useful tool to respond better to patients' needs. After concluding the initial pilot phase, HM has been structurally embedded in our care for HNC patients since April 2015.

### 2.2 First evaluation of HM: key lessons and mixed‐methods design

In the current report, we review the challenges we experienced during the initial implementation phase by sharing barriers and facilitators based on pragmatic experience in implementing HM initiative. Some of these impressions are briefly summarized in narrative and tabular form in the beginning of Section 3. In addition, to evaluate patient experiences, we conducted a mixed‐methods study using quantitative data from a self‐developed patient‐reported experience measures (PREM) questionnaire and qualitative data from interviews. Ethical approval was not necessary according to the Dutch Medical Research Involving Human Subjects Act, as this study evaluated standard care.

#### 2.2.1 Quantitative method

We invited n = 151 patients diagnosed with HNC between October 2014 and April 2016 to anonymously complete a survey on the care they received during follow‐up visits at our outpatient clinic. This number of patients invited was not based on a power analysis, but was determined by the number of patients actively using HM at that time. In the accompanying letter provided to patients, informed consent was requested to use the data anonymously for the purpose of this evaluation study. There was no overlap in participants between the pilot phase and this evaluation study.

Two groups were distinguished: (a) HNC patients that were diagnosed prior to implementation of HM (standard care group), and (b) HNC patients that were diagnosed after implementation of HM (HM care group). Patients in group 2 were treated more recently than those in group 1. No other patient characteristics than age and gender were available due to the anonymous response. In the standard care group symptoms were discussed during standard clinical encounters. In the HM care group, patients filled out ePRO questionnaires before their doctors visit and the results were directly discussed during the consultation.

We developed a 12‐item PREM questionnaire with a 4‐point Likert scale for both groups of patients. Six extra items were developed specifically for the HM care group, based on questions on functioning of HM raised in our research meetings and meetings with health care professionals. In this survey for HM patients (supplementary Table 1a), we asked about the burden of filling out HM questionnaires and about the congruity of items addressed in HM and experienced by patients themselves. Both groups of patients were asked to answer questions on doctor‐patient communication, their preparation to discuss their individual conditions, physicians' awareness of individual patient conditions and efforts made to improve these individual patient conditions (supplementary Table 1b). We also asked questions about the length of the consultation and asked patients to rate their subjectively experienced quality of care ranging between 1 and 10. A higher score means a higher experienced level of quality of care.

#### 2.2.2 Qualitative method

Structured interviews (N = 15) were conducted in 2016 during one full consultation day of one HNC surgeon at the outpatient clinic. Only patients who received HM care and were in the early follow‐up phase, several months after curative treatment, were approached to take part in the interviews. Patients were selected randomly from physician's consultation visits at our outpatient clinic. The interviews were held by a female senior researcher with PhD degree (MO). There was no relationship established with participants prior to start of the interview, nor did participants have knowledge of the interview. Interviews were held either directly after the consultation with the HNC surgeon or after the moment patients filled in the HM ePROs at our outpatient clinic prior to the visit with their HNC surgeon. All interviews were held at the outpatient clinic of the Erasmus Medical Center. All approached patients agreed to cooperate with the interview, and they gave oral informed consent to use the interview for the purpose of this evaluation study. Patients were interviewed on the added value of HM and on how they think of HM in general. A semi‐structured interview guide was made which consisted five key questions, all with an open character, followed by more elaborate questions to follow‐through on the subject (Table 2). The questions were asked in a fixed order for all the interviews.

**TABLE 2:** Interview guide for structured interviews

1. What are your general experiences with “Healthcare Monitor”?
2. Do you find the way of working with “Healthcare Monitor” a good thing? And if so or not, why?
3. “Healthcare Monitor” provides your doctor with direct insight into you symptoms. What is your opinion about this?
4. Do you experience added value by filling health care questionnaires upfront to your doctor s control visit? If so, what is the added value in your opinion?
5. Do you have any suggestions for us to further improve our working method with Healthcare Monitor?

#### 2.2.3 Data analyses

All statistical analyses were performed using SPSS version 22 (IBM Corp. Armonk, NY). The quantitative data from the nonrandomized retrospective survey were analyzed using descriptive statistics. To compare patients in the HM care group to those in the standard care group on items 1‐7 of the PREM questionnaire, statistical analysis of categorical data was performed by chi‐square test and analysis of continuous data by Students' *t*‐test. Only four patients in the standard care group did not fill out the complete survey. Since these data were missing not at random, we left the data out of the analysis by performing a complete case analysis. Logistic regression analysis, adjusted for age and gender, was performed to analyze differences in experienced quality of care (items 8‐12) between HM care and standard care. The answers that patients provided on the 4‐point Likert scale were dichotomized to “agree” and “disagree” for this purpose. *P*‐values of <.05 were considered significant.

The semi‐structured interviews were thematically analyzed by a trained senior researcher with no relationship with the patients (MO). Qualitative content analysis was used for analysis of the data and inductive categories were derived. Based on the answers provided by patients during the interviews, three themes were identified from the data and clearly presented in the results.

## 3 RESULTS

### 3.1 Key clinical impressions from the setup and implementation of HM

The implementation of an ePRO structure in clinical practice includes significant challenges.^4^, ^36^, ^37^ Our experienced barriers and facilitators are summed up in Table 3.

**TABLE 3:** Facilitators and barriers for implementation of “Healthcare Monitor” care

Facilitators of “Healthcare Monitor”
Patients can fill out the questionnaires via internet at the comfort of their homes.
Trained volunteers can assist patients with filling out the questionnaires before the doctor's visit.
Clinicians have real‐time access to the results, which are graphically displayed inside the hospital information system.
Clinicians capture a holistic view of the patient including both physical and psychosocial functioning.
Review of longitudinal ePRO reports is possible to identify the course of physical and psychosocial complaints and to compare patients individual results with their peer group.
Clinicians use the results to provide direct individual feedback to the patient on the need for supportive care.
Clinicians can identify critical issues earlier.
Better counseling of patients is possible leading to better quality of care.
Patients can actively participate in their own care which strengthens patient empowerment.
Barriers to use “Healthcare Monitor”
The implementation of HM consumes time and energy while the organization still runs all the other activities it has going.
Dedication and support is needed from every member of the team.
For successful implementation, it takes effort to motivate all health care providers, administrative employees, and technology providers.
Many workplace and organizational adjustments are necessary.
A sustainable and robust technical environment is necessary.
Adequate resources are necessary so that patients who screen positive subsequently will receive diagnostic or clinical evaluation, and referral to specialty care as needed.
The extra costs needed to implement HM care are not reimbursed by health insurers or government yet.
Evaluation of the work process as a whole is needed in order to adapt user needs.

First of all, common ground is needed. One must gain support from every member of the team, including health care providers, administrative employees, and technology providers. This task will take some effort, but we believe the common goal of improving patient care makes it worthwhile.

We found conducting a pilot phase to be very helpful in reducing any “teething problems.” As of January 2019, 1737 patients have taken part in HM from their day of diagnosis and routinely receive this care, with 95% patient compliance at intake and over 80% at the different moments of follow‐up.

In comparison with the literature regarding use of PROs in oncology settings, our compliance rates are uncommonly high.^4^ We believe that the way we integrated ePROs in regular patient care leads to this high compliance. Reported data of the patient are directly used for the benefit of the patient, and the online system facilitates a better preparation for clinical consultation given the opportunity to fill out ePROs when it suits the patient.

Illiteracy, not having access to internet, and advanced age are known barriers for participation of patients in an ePRO structure.^4^, ^23^ We chose to train our already available group of volunteers in assisting patients with filling out HM questionnaires. This role is important for the sustainability of HM since the volunteers encourage patients to fill out the questionnaires themselves by showing the ease of the system. An important group of vulnerable patients who generally do not have access to digital solutions and may have problems with phrasing their complaints is thus supported to take part in HM care.

The graphical display of HM results inside the hospital information system is also helpful. The course of symptoms can be identified and patients' individual results can be compared with their peer group.

However, we also experienced that the implementation of HM consumes time and energy. Organizational and workplace adjustments were necessary, and the close cooperation with health care and technology providers was essential. For example, all medical staff (including secretaries, nurses, doctors, and case managers) was trained in using and/or interpreting HM. A small renovation of our outpatient clinic was necessary to have a private space available for filling out HM questionnaires on the iPad. Furthermore, after 2 years, we had to hire a new secretary to manage the HM system working schedules of the volunteer group and to oversee that every patient fills in the HM questionnaires beforehand (either at home or at the clinic). The provision of a powerful IT network and the assurance of data safety was necessary in order to facilitate a more efficient and safe work process.

#### 3.1.1 Quantitative results from the mixed‐methods study

Within the HM group, 45 of 71 patients (63.4%) completed this survey, and 46 of 66 patients (69.7%) completed the survey in the standard care group. No information was available on reasons for declining participation. Both groups of patients showed comparable distribution of age and gender. Within the HM group, we found that 31 (69%) of patients completed the HM questionnaires at home, and 12 (27%) used the help of a volunteer to complete the HM questionnaires at the outpatient clinic. The time needed to complete HM questionnaires as perceived by patients was on average 19 minutes (SD 8 minutes). A majority of 41 patients (91.1%) indicated that this time was not too short nor too long. A minority of patients found the HM questionnaires unclear (n = 2, 4%) and irrelevant (n = 2, 4%). The reasons these patients mentioned were “they're asking me to answer the same questions using different wording 20 times in a row” and “I have to answer questions on pain management that are not related to my tongue cancer.”

In univariate analysis, no significant differences on any of the 17 items were seen between the two groups of patients (Table 4). In comparison with standard care, more patients indicated that the use of HM helped physicians to have a more complete picture of patients and to have a focus on their specific condition; however, these were nonsignificant results. Also the length of the consultation as perceived by patients was shorter when using HM, but this was also not a significant difference. Logistic regression analysis showed that age and gender were not significant predictors for the experienced quality of care in both groups (items 8‐12). The subjectively overall experienced quality of care (item 6) was equally high in both groups.

**TABLE 4:** Perceptions of quality of care among patients receiving standard care vs those receiving electronic patient‐reported symptom monitoring

		Standard care (n = 42)			“Healthcare Monitor” (n = 45)			*P* value^a^
		Mean (SD)/frequency		%	Mean (SD)/frequency		%	
1	Age (years)	67.5 (11.3)			63.7 (9.7)			.092
2	Gender	Male	30	71.4		34	75.6%	.740
		Female	12	28.6%		11	24.4%	
3	Did the doctor discuss your most common health complaints?	Yes	37	88.1%		43	95.5%	.636
		No	5	11.9%		2	4.5%	
4	Has the doctor taken action when it comes to treating your complaints?	Yes	34	80.9%		41	91.1%	.375
		No	8	19.1%		4	8.9%	
5	Did you miss topics during the consultation?	Yes	12	28.6%		10	22.2%	.243
		No	30	71.4%		35	77.8%	
5A	Did you miss a topic on symptom burden during the consultation?	Yes	34	81.0%		39	86.7%	.286
		No	8	19.0%		6	13.3%	
5B	Did you miss a topic on psychosocial distress during the consultation?	Yes	37	88.1%		39	86.7%	.621
		No	5	11.9%		6	13.3%	
5C	Did you miss a topic on comorbidity during the consultation?	Yes	40	95.2%		42	93.3%	.662
		No	2	4.8%		3	6.7%	
5D	Did you miss a topic on medication during the consultation?	Yes	40	95.2%		42	93.3%	.662
		No	2	4.8%		3	6.7%	
5E	Did you miss a topic on influence of disease on your partner during the consultation?	Yes	37	88.1%		40	88.9%	.748
		No	5	11.9%		5	11.1%	
5F	Did you miss a topic on influence of disease on your occupation during the consultation?	Yes	39	92.9%		43	95.6%	.645
		No	3	7.1%		2	4.4%	
5G	Did you miss a topic on influence of disease on your social life during the consultation?	Yes	39	92.9%		40	88.9%	.725
		No	3	7.1%		5	11.1%	
6	Subjective rating (1) to (10) of experienced quality of care	8.2 (SD 1.3)			8.1 (SD 1.1)			.695
7	Length of consultation (minutes)	12 (SD 6)			11 (SD 4)			.149
8	I felt well prepared for the consultation with my treating physician	Yes	41	97.6%		45	100%	.458
		No	0	0%		0	0%	
		Missing	1	2.4%		0	0%	
9	My treating physician was focused on my specific complaints	Yes	39	92.9%		42	93.3%	.398
		No	3	7.1%		2	4.4%	
		Missing	0	0%		1	2.3%	
10	My treating physician had a complete picture of me	Yes	30	71.4%		38	84.4%	.554
		No	8	19%		7	15.6%	
		Missing	4	9.6%		0	0%	
11	My treating physician paid attention to my specific complaints	Yes	37	88.1%		41	91.1%	.291
		No	5	11.9%		3	6.6%	
		Missing	0	0%		1	2.3%	
12	My treating physician undertook action in response to my specific complaints	Yes	33	78.6%		40	88.8%	.585
		No	4	9.5%		4	8.8%	
		Missing	5	11.9%		1	2.4%	

a Categorical data analyzed by chi‐square test, continuous data by Students' *t*‐test, equal variances not assumed.

#### 3.1.2 Qualitative results from the mixed‐methods study

The answers provided by patients during the interviews were categorized into three themes. The themes are (a) patient preparation for the consultation, (b) doctor‐patient communication, and (c) patient experience with HM care. A summary of the results including verbatim examples are provided in Table 5. Patients also shared their views of the pros and cons of HM and suggestions for improvement. (Table 6).

**TABLE 5:** Verbatim examples of three overall themes regarding patients' views of “Healthcare Monitor” experience in practice

Theme	Verbatim example^a^
Better *preparation* patient Patients see HM as a tool to be better prepared for their visit to the doctor. Patients forget less and because they have been through all the questions beforehand, they are more conscious on how they really feel.	“This system makes it easier for us as a patient to come to the doctor, because you have been through all the questions yourself.” “If I did not have this preparation with the questionnaires when going to my control visit, I would close the door after my consult thinking 'I should have asked my doctor this or that!” “It is good for me, as a patient, to fill out these questionnaires because I need to think myself how I really feel, so that I can ask my doctor the right questions.” “You already shared upfront (with the questionnaires) how you are doing”
Better doctor‐patient *communication* A good preparation to the doctor's visit generally contributes to the quality of the conversation and HM seems to play an important role in that process. Patients mentioned that the doctor can see at one glance how they are doing. The fact that the doctor has this complete overview of the patient in advance, makes it easier for them to speak to their doctor.	“When you fill out the questionnaires, I understand what the doctor wants to know from me, so I do not feel overwhelmed by the doctor as I know now what to expect.” “It is good that the doctor has a complete overview of how I am doing and how I cope with it.” “You can be more efficient in your talk with the doctor and it might result in shorter waiting times.” “I do not have to think so hard anymore (how I am doing) during the doctor's visit.”
Positive patient *experience* The way of working with HM contributes to a positive experience and a sense of security and peace. Patients are feeling heard, taken seriously, and they felt there is room to share both their physical and mental complaints.	“By filling out the questionnaires I feel that the doctor looks at me in a professional manner. There is a good overall control.” “The doctor can directly follow through at my complaints and that is a good thing.” “It is a nice feeling that they pay attention to me.” “I think the “Healthcare Monitor” is a good thing, especially sometime after treatment, when you reflect on the whole trajectory and how you feel about that.”

a All quotes are based on responses from varied patients.

**TABLE 6:** Patient perceptions of pros and cons of Healthcare Monitor and suggestions for improvement, from the structured interviews

Pros	Cons
I experience a more efficient doctors visit I love to fill out the questionnaires: it is easy and quick, so I am happy with it The *Healthcare Monitor* gives me insight in how I really feel I feel much better prepared! A big advantage is that I can fill outthe questionnaires at home via internet I feel a greater commitment of my doctor in this way I feel at ease with the help of a volunteer at the hospital when filling out the questionnaires It is very well organized in comparison to other hospitals	It is a fuss: so many questionnaires Questionnaires sometimes look alike Every time when I fill out the questionnaires, I feel confronted with the disease I had…
Suggestions for improvement It is important to emphasize that the “Healthcare Monitor” is beneficial for you as an individual patient. Would be better if questions are not only related to the last week, however, to the period in between the control visits.	

## 4 DISCUSSION

The overall aim of this study was to provide insight into our key lessons on the set‐up and implementation of HM and into how HNC patients experience and value HM care in clinical practice.

We believe that the implementation of HM included significant challenges and also had demonstrated to be a worthwhile investment. The results of the qualitative analyses look very promising. However, the quantitative analyses did not show any significant results. Quantitative analyses showed that HM users scored higher on some questions of the PREM questionnaire than those who had received standard care. HM users experienced more often that their physician had a complete picture of them and undertook action in response to their specific complaints. The differences in these percentages were not statistically significant but they exceeded 10 points and thus appear to be clinically meaningful results worthy of further research. No significant difference regarding the perceived quality of care could be found between groups possibly because of a ceiling effect. Also, the standard care patients were further along in their recovery than patients in the HM group, and this might have introduced confounding by time since treatment.

Qualitative analyses suggested that HNC patients who received HM care noticed more focus on critical issues and an increased efficiency of the consultation. Patients felt better prepared and were more conscious on how they really feel, and mentioned their clinicians had a more holistic view on their symptoms. Furthermore, structurally monitoring ePROs and discussing the results with individual patients contributed to a positive experience and a sense of security. These qualitative findings are in line with earlier (both qualitative and quantitative) studies on the value of using PROMs in HNC clinical practice to improve communication between doctors and patients and to facilitate screening of symptoms and psychological distress.^16^, ^22^, ^23^, ^35^ In our study, HNC patients also experienced a better doctor‐patient communication with HM.

The results from our evaluation indicate that HM might be useful in identifying the latent needs of patients. The ePRO questions stimulate patients to think about issues in relation to their disease they might not have thought about before. As a result, awareness of symptoms and a sense of the normal course of disease can be raised, leading to patient empowerment.

Besides the improvement of perceived patient care as suggested by the qualitative results, we learned that conducting scientific studies can benefit from the existing ePRO structure within HM as well. The logistic set‐up, including organizational and workplace adjustments, and the relationship of trust between patients and trained volunteers, creates opportunities to counsel patients on research projects. Longitudinal ePRO data can enhance benchmarking on an individual level in our clinic as well as in larger, multicenter, studies.

We would like to share our lessons with other HNC clinics, in order to improve patient care and create benchmark possibilities. Therefore, we prepared a guideline with eight questions that might be helpful to address before implementing an ePRO structure into daily clinical practice (see Table 7).

**TABLE 7:** Toolkit: with eight essential questions toward implementation of an ePRO structure in HNC clinics

Step 1: “Why do I want to measure outcome in this patient group?”
Step 2: “What are the right outcomes measures for this patient group?”
Step 3: “What questionnaires should I use, are those validated and readily available?”
Step 4: “What are the right moments to measure ePROs?”
Step 5: “Are there any workspace or organizational adjustments necessary?”
Step 6: “How do I take data‐integrity into account?”
Step 7: “What will I do with the obtained data?”
Step 8: “Do I have adequate resources available for patients in need of extra care?”

Abbreviations: ePROs, electronic patient‐reported outcome measures; HNC, head and neck cancer.

### 4.1 Future perspectives

Although we experienced that the implementation of HM took a lot of effort, the experienced value made it worthwhile. This positive balance contributed to a sustainable system. In order to maintain this balance, we believe two areas of focus for the future are important.

One way of achieving a lower experienced effort might be by exploring computer adaptive testing methods (CAT) in HM, since the use of traditional PROMs often requires patients to answer items that are not directly applicable to them.^38^, ^39^ CAT has several advantages including reduced patient burden and increased question relevance to individual patients.^40^ Another way HM might be improved is the optimization of the graphical display of the results. We are currently developing a dashboard including HM results, which can help physicians to efficiently get an overview of all data. In order to achieve a higher experienced value, research is needed to obtain more insight into the referral and uptake of supportive care services following the individual feedback from doctors to patients using HM.

An ePRO setup such as HM may also have direct influence on health care costs. On the one hand, one can imagine that when more symptoms (eg, psychosocial) are being recognized due to HM, this will also lead to more diagnostics or involvement of other health care providers (eg, psychiatrist), and therefore probably to higher costs. On the other hand, HM could also contribute to lower rather than higher costs due to earlier identification of conditions and reduced frequency of regular outpatient clinic visits. A cost‐effectiveness study seems appropriate, especially in the context of value‐based health care.

### 4.2 Study limitations

The mixed‐methods design we used for this study was sufficient as a first step and forms a useful foundation for further research.

However, this study has some limitations. Both the structured interviews and the retrospective nonrandomized survey have a small sample size. Although the questions of the structured interview all have an open character, they may have introduced a positive bias as some of the questions already include the suggestion of HM being beneficial. The small sample size of the quantitative analysis and absence of a power analysis might explain the nonsignificant results. Also, clinical characteristics or demographic variables other than age and gender were not available.

Another limitation of this study is that we did not evaluate the experience of health care providers on the use of HM. Therefore, we are currently preparing a yearly evaluation questionnaire for patients and professionals.

## 5 CONCLUSION

Our qualitative data suggest that integration of our ePRO clinical support system *Healthcare Monitor* into routine care for HNC patients may lead to increased patient‐centered care and an improved perception of doctor‐patient communication, and may enable a holistic approach, and enhance patient empowerment.

Structurally monitoring ePROs and discussing the results with each patient appears to contribute to a positive patient experience and facilitates screening and follow‐up of symptoms including psychological distress. However, future research is needed to analyze the potential benefits more extensively.

## Supporting information


**Appendix S1:** Supporting information

## References

[1] Basch E , Deal AM , Kris MG , et al. Symptom monitoring with patient‐reported outcomes during routine cancer treatment: a randomized controlled trial. J Clin Oncol. 2016;34(6):557‐565. 26644527 10.1200/JCO.2015.63.0830 PMC4872028

[2] Porter ME . What is value in health care? N Engl J Med. 2010;363(26):2477‐2481. 21142528 10.1056/NEJMp1011024

[3] Squitieri L , Bozic KJ , Pusic AL . The role of patient‐reported outcome measures in value‐based payment reform. Value Health. 2017;20(6):834‐836. 28577702 10.1016/j.jval.2017.02.003 PMC5735998

[4] Howell D , Molloy S , Wilkinson K , et al. Patient‐reported outcomes in routine cancer clinical practice: a scoping review of use, impact on health outcomes, and implementation factors. Ann Oncol. 2015;26(9):1846‐1858. 25888610 10.1093/annonc/mdv181

[5] Andikyan V , Rezk Y , Einstein MH , et al. A prospective study of the feasibility and acceptability of a web‐based, electronic patient‐reported outcome system in assessing patient recovery after major gynecologic cancer surgery. Gynecol Oncol. 2012;127(2):273‐277. 22871467 10.1016/j.ygyno.2012.07.124 PMC3641568

[6] Jensen RE , Snyder CF , Abernethy AP , et al. Review of electronic patient‐reported outcomes systems used in cancer clinical care. J Oncol Pract. 2014;10(4):e215‐e222. 24301843 10.1200/JOP.2013.001067 PMC4094646

[7] Hilarius DL , Kloeg PH , Gundy CM , Aaronson NK . Use of health‐related quality‐of‐life assessments in daily clinical oncology nursing practice: a community hospital‐based intervention study. Cancer. 2008;113(3):628‐637. 18543317 10.1002/cncr.23623

[8] Velikova G , Booth L , Smith AB , et al. Measuring quality of life in routine oncology practice improves communication and patient well‐being: a randomized controlled trial. J Clin Oncol. 2004;22(4):714‐724. 14966096 10.1200/JCO.2004.06.078

[9] Pakhomov SV , Jacobsen SJ , Chute CG , Roger VL . Agreement between patient‐reported symptoms and their documentation in the medical record. Am J Manag Care. 2008;14(8):530‐539. 18690769 PMC2581509

[10] Velikova G , Keding A , Harley C , et al. Patients report improvements in continuity of care when quality of life assessments are used routinely in oncology practice: secondary outcomes of a randomised controlled trial. Eur J Cancer. 2010;46(13):2381‐2388. 20570138 10.1016/j.ejca.2010.04.030

[11] Valderas JM , Kotzeva A , Espallargues M , et al. The impact of measuring patient‐reported outcomes in clinical practice: a systematic review of the literature. Qual Life Res. 2008;17(2):179‐193. 18175207 10.1007/s11136-007-9295-0

[12] Chen J , Ou L , Hollis SJ . A systematic review of the impact of routine collection of patient reported outcome measures on patients, providers and health organisations in an oncologic setting. BMC Health Serv Res. 2013;13:211. 23758898 10.1186/1472-6963-13-211 PMC3700832

[13] Basch E , Jia X , Heller G , et al. Adverse symptom event reporting by patients vs clinicians: relationships with clinical outcomes. J Natl Cancer Inst. 2009;101(23):1624‐1632. 19920223 10.1093/jnci/djp386 PMC2786917

[14] Gotay CC , Kawamoto CT , Bottomley A , Efficace F . The prognostic significance of patient‐reported outcomes in cancer clinical trials. J Clin Oncol. 2008;26(8):1355‐1363. 18227528 10.1200/JCO.2007.13.3439

[15] Basch E . Patient‐reported outcomes ‐ harnessing patients' voices to improve clinical care. N Engl J Med. 2017;376(2):105‐108. 28076708 10.1056/NEJMp1611252

[16] Kotronoulas G , Kearney N , Maguire R , et al. What is the value of the routine use of patient‐reported outcome measures toward improvement of patient outcomes, processes of care, and health service outcomes in cancer care? A systematic review of controlled trials. J Clin Oncol. 2014;32(14):1480‐1501. 24711559 10.1200/JCO.2013.53.5948

[17] Coyne JC . Benefits of screening cancer patients for distress still not demonstrated. Br J Cancer. 2013;108(3):736‐737. 23370207 10.1038/bjc.2013.16 PMC3593562

[18] Palmer SC , van Scheppingen C , Coyne JC . Clinical trial did not demonstrate benefits of screening patients with cancer for distress. J Clin Oncol. 2011;29(10):e277‐e278. author reply e9‐80. 21300932 10.1200/JCO.2010.34.1206

[19] Carlson LE , Waller A , Groff SL , Zhong L , Bultz BD . Online screening for distress, the 6th vital sign, in newly diagnosed oncology outpatients: randomised controlled trial of computerised vs personalised triage. Br J Cancer. 2012;107(4):617‐625. 22828610 10.1038/bjc.2012.309 PMC3419958

[20] Mitchell AJ . Screening for cancer‐related distress: when is implementation successful and when is it unsuccessful? Acta Oncol. 2013;52(2):216‐224. 23320770 10.3109/0284186X.2012.745949

[21] Funk R , Cisneros C , Williams RC , Kendall J , Hamann HA . What happens after distress screening? Patterns of supportive care service utilization among oncology patients identified through a systematic screening protocol. Support Care Cancer. 2016;24(7):2861‐2868. 26838023 10.1007/s00520-016-3099-0

[22] Krebber AM , Jansen F , Cuijpers P , Leemans CR , Verdonck‐de Leeuw IM . Screening for psychological distress in follow‐up care to identify head and neck cancer patients with untreated distress. Support Care Cancer. 2016;24(6):2541‐2548. 26694718 10.1007/s00520-015-3053-6 PMC4846709

[23] Rogers SN , Lowe D , Lowies C , et al. Improving quality of life through the routine use of the patient concerns inventory for head and neck cancer patients: a cluster preference randomized controlled trial. BMC Cancer. 2018;18(1):444. 29669529 10.1186/s12885-018-4355-0 PMC5907378

[24] Cohen EE , LaMonte SJ , Erb NL , et al. American Cancer Society Head and Neck Cancer Survivorship Care uideline. CA Cancer J Clin. 2016;66(3):203‐239. 27002678 10.3322/caac.21343

[25] De Boer MF , McCormick LK , Pruyn JF , Ryckman RM , van den Borne BW . Physical and psychosocial correlates of head and neck cancer: a review of the literature. Otolaryngol Head Neck Surg. 1999;120(3):427‐436. 10064650 10.1016/S0194-5998(99)70287-1

[26] Weymuller EA Jr , Bhama PK . Quality of life in head and neck cancer patients. Expert Rev Anticancer Ther. 2007;7(9):1175‐1178. 17892417 10.1586/14737140.7.9.1175

[27] Offerman MP , Pruyn JF , de Boer MF , Busschbach JJ , Baatenburg de Jong RJ . Psychosocial consequences for partners of patients after total laryngectomy and for the relationship between patients and partners. Oral Oncol. 2015;51(4):389‐398. 25631352 10.1016/j.oraloncology.2014.12.008

[28] Aaronson NK , Ahmedzai S , Bergman B , et al. The European Organization for Research and Treatment of cancer QLQ‐C30: a quality‐of‐life instrument for use in international clinical trials in oncology. J Natl Cancer Inst. 1993;85(5):365‐376. 8433390 10.1093/jnci/85.5.365

[29] Bjordal K , Hammerlid E , Ahlner‐Elmqvist M , et al. Quality of life in head and neck cancer patients: validation of the European Organization for Research and Treatment of Cancer Quality of Life Questionnaire‐H&N35. J Clin Oncol. 1999;17(3):1008‐1019. 10071296 10.1200/JCO.1999.17.3.1008

[30] Zigmond AS , Snaith RP . The hospital anxiety and depression scale. Acta Psychiatr Scand. 1983;67(6):361‐370. 6880820 10.1111/j.1600-0447.1983.tb09716.x

[31] Belafsky PC , Mouadeb DA , Rees CJ , et al. Validity and reliability of the eating assessment tool (EAT‐10). Ann Otol Rhinol Laryngol. 2008;117(12):919‐924. 19140539 10.1177/000348940811701210

[32] Jacobsen B , Johnson A , Grywalski C , et al. The voice handicap index (VHI): development and validation. Am J Speech‐Lang Pathol. 1997;6:66‐70.

[33] EuroQol G . EuroQol–a new facility for the measurement of health‐related quality of life. Health Policy. 1990;16(3):199‐208. 10109801 10.1016/0168-8510(90)90421-9

[34] Scott NW , Fayers PM , Aaronson NK , et al. EORTC QLQ‐C30 Reference Values. Brussels, Belgium: EORTC Quality of Life Group; 2008.

[35] Krebber AH , van Uden‐Kraan CF , Melissant HC , et al. A guided self‐help intervention targeting psychological distress among head and neck cancer and lung cancer patients: motivation to start, experiences and perceived outcomes. Support Care Cancer. 2017;25(1):127‐135. 27585808 10.1007/s00520-016-3393-x PMC5127860

[36] Antunes B , Harding R , Higginson IJ , Euroimpact . Implementing patient‐reported outcome measures in palliative care clinical practice: a systematic review of facilitators and barriers. Palliat Med. 2014;28(2):158‐175. 23801463 10.1177/0269216313491619

[37] Boyce MB , Browne JP , Greenhalgh J . The experiences of professionals with using information from patient‐reported outcome measures to improve the quality of healthcare: a systematic review of qualitative research. BMJ Qual Saf. 2014;23(6):508‐518. 10.1136/bmjqs-2013-002524 24505110

[38] Petersen MA , Aaronson NK , Arraras JI , et al. The EORTC CAT Core‐the computer adaptive version of the EORTC QLQ‐C30 questionnaire. Eur J Cancer. 2018;100:8‐16. 29936066 10.1016/j.ejca.2018.04.016

[39] Wainer H . Computerized Adaptive Testing: a Primer. New York, NY: Lawrence Erlbaum Associates; 2000.

[40] Reise SP . Handbook of Item Response Theory Modeling: Applications to Typical Performance Assessment. New York, NY: Routledge; 2015.

