# Supplementary material for: Keys to successful implementation of routine symptom monitoring in head and neck oncology with “Healthcare Monitor” and patients' perspectives of quality of care
Source: Head Neck. 2020 Aug 18;42(12):3590–600. doi: 10.1002/hed.26425 (PMC7754276; doi:10.1002/hed.26425)
Supplement: Supplementary file 1 — Appendix S1: Supporting information [file HED-42-3590-s001.docx]

**Supplementary table 1a:** PREM questionnaire – 6 items on HM care

1) Where did you fill out the Healthcare Monitor questionnaires?

- At home, via internet
- At the outpatient clinic, using an iPad WITHOUT assistance by a volunteer
- At the outpatient clinic, using an iPad WITH assistance by a volunteer

2) How much time does it take to fill out the questionnaires? …………………….minutes.

3) Filling out the questionnaires:

- Took too long
- Took too short
- Was just right in terms of time

4) Were there any questions unclear in the Healthcare Monitor questionnaires?

- Yes, namely: ………………………………………………………………………..
- No:

5) Were there any questions in the Healthcare Monitor that did not apply to you?

- Yes, namely: ………………………………………………………………………..
- No:

6) Did you miss any questions in the Healthcare Monitor? (several answers are possible)

- yes, about the presence of (an) other disease(s) that I have
- yes, about the medication I use
- yes, about the impact of the disease on my partner / relative(s)
- yes, about the impact of the disease on the loss of employment
- yes, about: ……………………………………………
- no

**Supplementary table 1b:** PREM questionnaire – 12 items for both groups of patients

1) What is your gender? Male/female

2) What is your age? ….. years

*For the following questions, keep the last control visit in mind:*

3) Did the doctor discuss your most common health complaints? Yes/no

4) Has the doctor taken action when it comes to treating your complaints? Yes/no

5) Did you miss topics during the consultation? (several answers are possible)

- yes, about the functional complaints that I have (eg problems with the voice or swallowing)
- yes, about the psychosocial complaints that I have
- yes, about the presence of (an) other disease(s) that I have
- yes, about the medication I use
- yes, about the impact of the disease on my partner / relative(s)
- yes, about the impact of the disease on the loss of employment
- yes, about: ……………………………………………
- no

6) If you give a score (between "0" and "10") about the follow-up on your complaints during the consultation with the doctor, which rate do you give?

Report rating:………………………………………………….

7) How much time on average does the consultation with the doctor take: …………………. minutes.

**Think back to the last visit to your ENT doctor at the outpatient clinic. Would you like to fill in the following questions by circling the correct answer for each question?**

|  |  | **Totally agree** | **Agree** | **Disagree** | **Totally disagree** |
| --- | --- | --- | --- | --- | --- |
| 8. | I felt well prepared for the visit to my doctor | 1 | 2 | 3 | 4 |
| 9. | The conversation with my doctor was focused on the complaints I experience | 1 | 2 | 3 | 4 |
| 10. | My doctor got a complete picture of me during the conversation | 1 | 2 | 3 | 4 |
| 11. | My doctor showed attention for my complaints during the conversation | 1 | 2 | 3 | 4 |
| 12. | My doctor took action in response to my complaints (for example: medication, referral to another specialist) | 1 | 2 | 3 | 4 |

Remarks and / or recommendations regarding the consultation with my ENT specialist at the outpatient clinic? ………………………………………………………………………………………………………………………………………………………………………………………………………………………………………………………………………………………………………………………………………………………………………………………………………………………………………………………………………………………
